# Associations between genetic HPV 16 diversity and cervical cancer prognosis

**DOI:** 10.1371/journal.pone.0308895

**Published:** 2025-06-25

**Authors:** Patrícia Patury, Fabio B. Russomano, Miguel Angelo Martins Moreira, Nádia R. C. Kappaun, Liz Maria de Almeida, Raquel B. M. Carvalho, Luís Felipe. L. Martins

**Affiliations:** 1 Gynecologic Oncology Department, Brazilian National Cancer Institute (INCA), Ministry of Health, Rio de Janeiro, Rio de Janeiro, Brazil; 2 Department of Clinical and Surgical Care of Women, National Institute of Women, Children and Adolescents Health Fernandes Figueira, Rio de Janeiro, Rio de Janeiro, Brazil; 3 Tumoral Genetics and Virology Program, Brazilian National Cancer Institute (INCA), Ministry of Health, Rio de Janeiro, Rio de Janeiro, Brazil; 4 Department of Physiotherapy, Brazilian National Cancer Institute (INCA), Ministry of Health, Rio de Janeiro, Rio de Janeiro, Brazil; 5 Division of Surveillance and Situation Analysis, Brazilian National Cancer Institute (INCA), Ministry of Health, Rio de Janeiro, Rio de Janeiro, Brazil; 6 Department of Female Endocrinology, National Institute of Women, Children and Adolescents Health Fernandes Figueira, Rio de Janeiro, Rio de Janeiro, Brazil; Ruđer Bošković Institute: Institut Ruder Boskovic, CROATIA

## Abstract

**Introduction:**

Cervical cancer (CC) arises as a result of chronic and persistent female infection by different oncogenic human papillomaviruses (HPV). The incidence of this disease is still high in developing countries, such as Brazil, where diagnosis is often made in advanced stages. HPV 16 is the most common Papillomavirus genotype in CC worldwide. Studies regarding the association of different HPV 16 lineages with overall and disease-free CC survival rates can contribute to further understanding the behavior of different HPV 16 lineages concerning the prognosis of CC cases.

**Objective:**

To assess the CC prognosis of patients treated in a Brazilian institution concerning HPV16 lineages.

**Methods:**

Data were obtained from a prospective cohort of 334 patients with CC recruited between July 2011 and March 2014 and treated at the Brazilian National Cancer Institute (INCA), in Rio de Janeiro, Brazil. HPV 16 lineages were identified in tumor tissue samples. Genetic HPV 16 diversity comprised 218 cases of lineage A, 10 of lineage B, 10 of lineage C, and 96 of lineage D. In addition to HPV 16 lineages, age, histopathological type, staging, and treatment completion were evaluated as predictors of CC prognosis.

**Results:**

The median patient age was 48 years. The most common histopathological type was squamous cell carcinoma (82.3%), followed by adenocarcinoma. Locally advanced disease staging was the most frequently detected, represented by similar stage II and III percentages (36.2% and 37.7%), followed by initial stage I (19.2%) and stage IV (6.9%). Two hundred two patients completed CC treatment. Age, histological type, staging, and treatment completion were associated with a higher risk of death, which was not observed for the HPV 16 lineage variable. With regard to age, an increase in each year of life led to approximately a 1% increase in the risk of death. Other histopathological types (poorly differentiated carcinoma, adenosquamous, neuroendocrine, and sarcoma) were associated with a higher risk of death compared with adenocarcinoma. Patients diagnosed in advanced stages exhibited a higher risk of death, and those who did not complete treatment exhibited an over 2-fold increased risk of death.

**Conclusion:**

This study found no associations between HPV 16 lineages A, B, C, and D and CC prognosis.

## Introduction

Cervical cancer (CC) remains a serious public health problem, especially in less developed countries, despite being considered a preventable disease through vaccination, screening, and treatment of precursor lesions. In countries with an opportunistic screening system, such as Brazil, or in those without public screening programs, CC diagnoses are often made when women already present with advanced symptoms, compromising survival and quality of life [1].

Cervical cancer is the third most common type of cancer in Brazil among women, excluding non-melanoma skin cancer. The number of new CC cases expected in Brazil is 17,010 for each year of the 2023–2025 triennium, with a mortality rate of 6,627 deaths noted for 2021 [2,3].

CC evolution in women with similar prognostic factors has not been fully clarified. Age, staging at diagnosis, lymphovascular space invasion, histopathological type, and presence of anemia are classically associated with worse CC prognoses [1,4]. The prognostic value of oncogenic human papillomavirus (HPV) DNA detection in tumors has not yet been established, and studies with this objective have reported conflicting results. Some authors postulate that the presence of oncogenic HPV may be a useful CC prognostic marker, as this makes the progression of precursor lesions more aggressive [5,6]. Others, however, stated that different types of oncogenic HPV have no prognostic value in patients with CC [7,8].

HPV 16 is most commonly associated with CC, and its four lineages were initially named according to the geographic region where they were most frequently identified and associated with local ethnicities [9,10]. Since 2013, however, they have been renamed A, B, C, and D [11–14]. Each HPV 16 lineage is subdivided into sublineages as follows: A1-A3 (corresponding to European and Asian lineages), A4 (corresponding to Asian lineage); B1-B4 (corresponding to African lineage 1); C1-C4 (corresponding to African lineage 2); D1-D3 (corresponding to North American and Asian-American lineages) [9,10].

A study has indicated that various lineages of HPV16 may exhibit distinct pathogenic profiles, with certain lineages demonstrating a significantly higher association with an increased risk of CC development compared to others. This differential pathogenicity underscores the importance of lineage-specific analysis in understanding the oncogenic potential of HPV16 and its implications for cervical cancer prevention and treatment strategies [15]. Some authors have demonstrated that lineages B, C, and D play a more important role in the progression of precursor cervix lesions in invasive CC then lineage A [9,16,17]. However, other studies have demonstrated that the D lineage is particularly highly associated with viral infection persistence and progression to CC [18–20].

Regarding the prognostic value of different HPV16 lineages and sublineages in CC cases, Tornesello *et al.* [21] suggested that the AA (Asian-American) lineage was associated with more aggressive CC. Another study evaluated 301 *in situ* cases and 727 CC cases and reported a higher frequency of HPV 16 D3 and A4 in CC cases and lower disease-free survival rates in women infected with the HPV 16 B lineage compared with lineages A, C, and D [22]. In another assessment, Zuna *et al.* [23] reported more advanced stages (II-IV) and lower survival rates in women infected with the European HPV 16 lineage among 155 patients with CC.

Given these contradictions and the possibility that differences may be associated with population characteristics or influenced by treatment strategies, this study aimed to assess whether the HPV 16 lineages present in CC cases in Rio de Janeiro, Brazil, influence CC prognosis regarding overall survival and progression-free survival.

## Methods

### Patients

This prospective cohort study was conducted at a specialized gynecological oncology center at the Brazilian National Câncer Institute (INCA), from July 2011 to March 2014. The study selected women aged 18 years or older, diagnosed with FIGO-2009 IB1 or higher cervical cancer (CC), and excluded patients who had undergone previous cancer treatment [17,24]. All participants were monitored over the course of treatment and over five-year period after treatment completion, and their clinical data were collected until January 2022. All participants provided written informed consent before participation. Ethical approval for the study was provided by the INCA Ethics and Research Committee (number 156/10, on 02/25/2011) and a specific amendment for this study (CAAE: 53398416.0.0000.5274, on 03/01/2016). All patients signed a consent form.

After signing the informed consent and allowing the collection of a new tumoral biopsy, a fresh tumoral biopsy was collected from each woman and immediately immersed in RNAlater (Thermo Fisher), stored for 24 hours at 4°C, then stored at −80°C, and deposited in the Brazilian National Cancer Institute Tumor Bank. Total DNA was isolated with the QIAamp DNA Mini Kit (Qiagen®). The identification of HPV genotypes and HPV16 lineages was performed following the methodologies described in Almeida et al. [24] and Vidal et al. [17], respectively. Briefly, HPV detection was performed by PCR amplification using primer sets PGMY07 and PGMY09 [25], reactions without PCR products were submitted to a nested PCR with primers GP5 + /GP6+ [26]. 

Nine hundred sixty-eight women with a histopathological diagnosis of CC were enrolled at the hospital during the research period. The HPV types and lineages in positive HPV16 cases were identified by tumor tissue biopsies obtained at the first consultation for 590 (60.9%) patients who gave their consent. New samples were not obtained for the remaining cases due to technical difficulties, poor clinical conditions or refusal to undergo the procedure.

### DNA isolation and HPV16 genotypes and lineages identification

Total DNA was isolated using a QIAamp DNA Mini Kit (Qiagen®). The HPV genotype and HPV16 lineage identification were previously described [17,24]. Briefly, HPV detection was performed by PCR amplification using primer sets PGMY07 and PGMY09 [25], reactions without PCR products were submitted to nested PCR with primers GP5 + /GP6+ [26]. To detect a possible PCR contamination in the nested PCR, an aliquot of the PCR control reaction (blank reaction) of the first PCR round (with PGMY07/PGMY09), carried out without DNA templates, was submitted to the round of amplification with GP5 + /GP6 + ; an additional blank reaction with GP5 + /GP6 + was also carried out. The absence of PCR products in all three blank reactions was considered that there was no contamination by a DNA sample or by a product of the first PCR reaction.

The amplified PCR products were purified using the GFX PCR and DNA Band Purification kit (GE Healthcare), sequenced with the Big Dye Terminator v3.1 Cycle Sequencing Kit (Applied Biosystems, Foster City, CA), and analyzed using an ABI Prism. 3130XL Genetic Analyzer (Applied Biosystems). All sequences were edited and analyzed using the 4Peaks Software (Nucleobytes, Amsterdam, Netherlands). HPV genotypes were identified using the Basic Local Alignment Search Tool (BLASTn) [17,27].

The presence of overlapping sequence peaks in Sanger sequencing indicated multiple infections. The identification of the different HPV genotypes for these samples was carried out with the High + Low Papillomastrip Kit (OPERON®), wich allows the identification of 37 HPV types, 6, 11, 16, 18, 26, 31, 33, 35, 39, 40, 42, 43, 44, 45, 51, 52, 53, 54, 56, 58, 59, 61, 62, 66, 67, 68, 69, 70, 71, 72, 73, 74, 81, 82, 83, 84, and 91. The data from women with multiple HPV infection were excluded in the analysis. Details regarding multiple infection findings are provided in Almeida *et al.* [24].

The identification of HPV16 lineages in these samples is detailed in Vidal et al [17]. Briefly, the *LCR* (Long Control Region) and *E6* genomic regions of HPV16 were amplified by PCR in two overlapping fragments encompassing approximately 1310 bp (base pairs). The PCR fragments were submitted to Sanger sequencing and lineage identification, as previously described [28], by identifying the presence of nucleotide variants in specific nucleotide positions. The lineage nomenclature adopted in the present work follows Burk *et al.* [14], considering the four lineages (A, B, C, and D) recognized for HPV16. Sequencing data for *LCR* and E6 were deposited in GenBank [29] (PopSet 970757329, with haplotypes and sequences corresponding to accession numbers KP965018.1 to KP965162.1).

Biopsies presenting HPV16 genotypes (n = 392) were submitted to PCR for amplification of the viral genomic regions *E6* and *LCR*, followed by DNA sequencing. This allowed the identification of different haplotypes and HPV16 lineages, which were previously reported by Vidal *et al*. [17].

**Fig 1:**
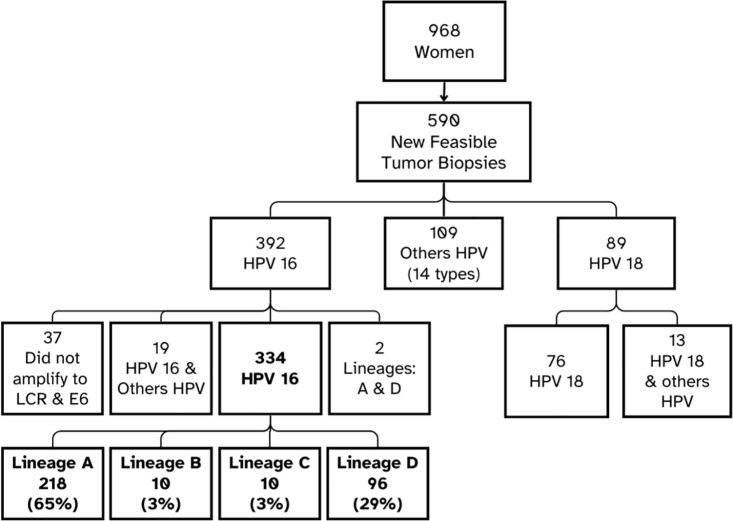
Flowchart depicting patient inclusion and types of detected HPV. Developed by the author.

Among the 392 HPV16-positive women, 19 exhibited co-infections with additional HPV genotypes, while two presented biopsies harboring two distinct HPV16 lineages (A and D in both cases). Additionally, PCR amplification of the LCR and E6 regions failed in 37 cases. Consequently, data from these women (n = 58) were excluded from subsequent analyses (Fig 1).

### Socioeconomic and clinical data

A questionnaire was used to obtain educational, environmental, reproductive, and socioeconomic characteristics of all enrolled patients. Clinical information concerning treatment and follow-up of patients infected with HPV16 was obtained from both physical and electronic records.

A completion of treatment was considered in those cases which all indicated treatments were applied. The necessity for adjuvant treatment in early-stage cases was determined when surgical intervention was performed, and the histopathological report indicated an intermediate or high risk of tumor recurrence. Complete treatment was established when complementary therapies were administered with full-dose chemotherapy and pelvic radiotherapy. In advanced-stage cases (stages II, III, and IV), treatment completeness was defined by the administration of complete doses of radiotherapy and brachytherapy in conjunction with chemotherapy.

Treatment was considered complete when the average dose of external radiotherapy delivered to the pelvis ranged from 45 to 50 Gy, applicable to both locally advanced disease treated with radiotherapy, brachytherapy, and concomitant chemotherapy, as well as in cases requiring external pelvic radiotherapy following surgical intervention [30]. In cases indicated for brachytherapy, treatments were considered complete when a dose of up to 10 Gy was administered [30–32]. The chemotherapy regimen was considered complete when five cycles of weekly cisplatin doses at 40 mg/m² were delivered during exclusive external pelvic radiotherapy and when chemotherapy was administered as adjuvant therapy following surgical treatment [33,34].

Disease persistence, whether complete or incomplete treatment, was defined in this study as the presence of loco-regional disease observed during gynecological examinations or the emergence of clinical symptoms that prompted imaging evaluation, subsequently confirming disease progression involving lymph nodes or distant organs. Tumor absence was assessed by clinical history and gynecological examination. In patients with doubt regarding persistence, computed tomography scans of the pelvis and abdomen were requested, as magnetic resonance imaging is not easily available in our hospital. Recurrence was characterized by the return of the disease in patients who initially achieved tumor remission after treatment. Disease-free survival was calculated exclusively for women who did not exhibit disease progression.

Regarding HPV16 lineages, it was opted to analyze lineages B and C collectively, as they are less prevalent, within a single category.

### Statistical analyses

The crude and adjusted prevalence ratios and their respective 95% confidence intervals (95% CI) were calculated to assess the potential association between HPV lineages and the variables under investigation. Variables such as staging, histological type, and treatment completion are directly linked to the prognosis of cervical cancer [35,36]. Given that staging and treatment completion were categorized into two distinct groups, a Poisson regression model with robust variance was used for the analysis. Models presenting p values < 0.05 were considered significant.

Kaplan-Meyer curves were used in a univariate and stratified manner for confounding variables in the survival analyses. The semiparametric proportional risk model (Cox model) was used for the multivariate analysis, and associations were considered significant when p < 0.05.

Overall survival rates were calculated by setting the survival time as the interval between the data of histopathological revision of the biopsy for diagnosis confirmation and the date of death or last follow-up. Initially, we used the Cox model to estimate the hazard ratio of the explanatory variables (HPV 16 lineage, age, histological type, staging, treatment completion) in relation to overall death in 5 years. The recurrence time was defined in months, considering the interval between the date of the end of the treatment and the date of the recurrence or the date of the last consultation with no signs of CC.

## Results

Patient and tumor characteristics are presented in Table 1. The median patient age was 48 years. Most women carried lineages A (65.3%) and D (28.7%), whereas lineage B was detected in 3% of all cases, and lineage C in another 3%.

**Table 1:** Description of the study population (n = 334).

Characteristics	n	%
***Sociodemographic***		
**Age (years)**		
Up to 39	96	28.7
40 - 49	93	27.8
50 - 64	106	31.7
65 and over	39	11.7
**Years of study**		
None	19	5.7
1 - 3	70	21.0
4 - 7	108	32.3
8 - 10	82	24.6
11 and over	55	16.5
**Marital status**		
Single	28	8.4
Married	184	55.1
Divorced	71	21.3
Widow	51	15.3
**Ethno-Racial Characteristics**		
White	108	32.3
Brown (“Parda”)	178	53.3
Black	46	13.8
Yellow (Asian descent)	2	0.6
***HPV 16 genotyping (detected lineages)***		
A	218	65.3
B	10	3.0
C	10	3.0
D	96	28.7
***Tumor and treatment features***		
**Histological Type**		
Squamous cell carcinoma	275	82.3
Adenocarcinoma	43	12.9
Others^1^	16	4.8
**Staging**		
I	64	19.2
II	121	36.2
III	126	37.7
IV	23	6.9
**Treatment completion**		
Yes	202	60.5
No	132	39.5
***Outcome***		
**Persistence**		
Yes	142	42.5
No	192	57.5
**Relapse (concerning 192 patients with no disease persistence)**		
Yes	68	35.4
No	124	64.6
**Death (considering the 334 patients)**		
Yes	194	58.1
No	140	41,9

^1^ 6 G3 Carcinoma cases, 5 Adenosquamous cases, 4 Neuroendocrine cases and 1 Sarcoma case.

The most common histopathological type was squamous cell carcinoma (82.3%), followed by adenocarcinoma (12.9%). Stages II and III were the most common (36.2% and 37.7%), followed by stage I (19.2%) and stage IV (6.9%). Only 202 women completed treatment aão nd 132 were unable to complete treatment due to a decline in clinical status related to the advanced stage of the disease. Regarding treatment results, 42,5% of the patients exhibited disease persistence, and among 192 (57.5%) patients with no CC signs, 68 (35.4%) relapsed. After the follow-up period, 140 out of the 334 (41,9%) patients were alive (see Table 1).

### Overall survival

The overall survival analysis was performed in association with HPV 16 lineages. Lineages B and C were grouped because of their low frequency (10 cases each). Lineages A and D were represented by 218 and 96 cases, respectively. The median follow-up time was 35.9 months (1Q = 12.6 months; 2Q = 36.3 months and 3Q = 66 months), and the median overall survival time was 40.3 months (CI_95%_: 29.9–49.8).

**Fig 2:**
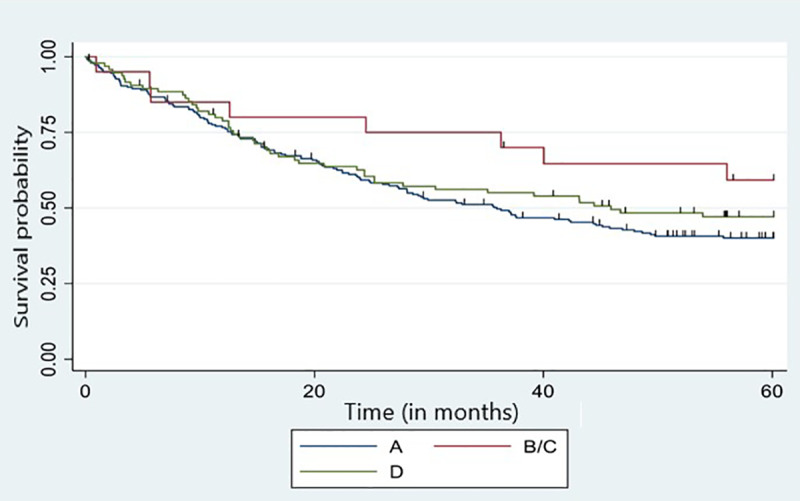
Five-year overall survival curve of cervical cancer patients since the beginning of treatment according to HPV 16 lineage*. Lineage A = 218 cases, B/C = 20 cases, D = 96 cases. *HPV 16 lineages B and C were grouped due to their low frequency.

Kaplan-Meyer curves according to each HPV 16 lineage are displayed in Fig 2. The median overall survival was 35.9 months among patients carrying lineage A and 45.9 months among those carrying lineage D. No estimated median was determined for the B/C group given that the survival curves were influenced by sample size. No significant difference was found between patients carrying tumors with distinct HPV16 lineages (p = 0.21; Log Rank test).

Table 2 shows the risk of patient death according to clinicopathological variables. Age, histological type, treatment completion, and staging were associated with a higher risk of death, whereas HPV 16 lineage was not. Regarding age, a 1% increase in the risk of death was noted for each year of life increase at diagnosis.

**Table 2:** Overall risk of death of patients with cervical cancer over a 5-year period (n = 334).

Characteristic	n	Crude HR^*^	P value
		(CI 95%)	
**HPV 16 lineage**			
A	218	1.18 (0.85–1.64)	0.32
B/C	20	0.67 (0.32–1.41)	0.29
D	96	1	–
**Age (years)**		1.01 (1.00–1.02)	0.02
**Histological type**			
Squamous cell carcinoma	275	1.51 (0.94–2.43)	0.09
Adenocarcinoma	43	1	–
Others	16	2.47 (1.17–5.19)	0.02
**Staging**			
I or II	185	1	–
III or IV	149	2.69 (2.00–3.61)	< 0.001
**Treatment completion**			
Yes	202	1	
No	132	5.71 (4.21–7.71)	< 0.0001

* univariate cox regression model hazard ratio (HR).

Tumors characterized as “others” histopathological types (poorly differentiated carcinoma, adenosquamous, neuroendocrine and sarcoma) were associated with a higher risk of death compared with adenocarcinoma (HR: 2.47) (95% CI 1.17–5.19). Patients with squamous cell carcinomas also exhibited a higher risk of death than those with adenocarcinomas, although this difference was not significant (Table 2).

Women diagnosed in advanced stages displayed a higher risk of death (HR: 2.69; CI: 2.00–3.61) and those who did not complete treatment presented more than five-fold increased risk of death (HR: 5.71; CI: 4.21–7.71), as shown in Table 2.

Considering the possibility that different HPV 16 lineages could be associated with variables that could lead to worse CC prognoses, prevalence ratios between HPV 16 lineages and other variables explaining worse prognosis were calculated (Table 3). The association between HPV 16 lineage A and staging reached borderline significance (p = 0.07). A significant association was observed between treatment completion and lineages B and C (p = 0.03).

**Table 3:** Prevalence ratios of HPV 16 lineages and explanatory variables for worse prognosis.

Characteristic	Advanced staging^1^		Histological type^2^		Did not finish treatment	
	Prevalence ratio*	p value	Prevalence ratio*	p value	Prevalence ratio*	p value
		(CI 95%)		(CI 95%)		(CI 95%)
**HPV 16 lineage**						
A	1.30 (0.97–1.74)	0.07	0.70 (0.35–1.38)	0.30	0.96 (0.72–1.28)	0.75
B/C	0.82 (0.40–1.67)	0.54	1.86 (0.52–6.73)	0.34	0.23 (006–0.89)	0.03
D	1				1	–

^1^Staging III and IV.

^2^Histological type (Squamous cell carcinoma, Adenocarcinoma, others); using the multiple logistic regression model.

### Disease-free survival

**Fig 3:**
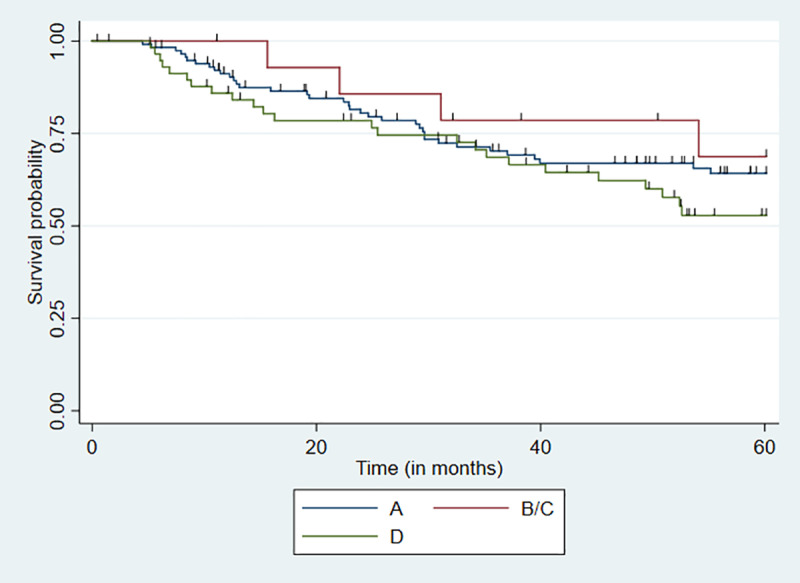
Disease-free survival curve in 5 years of patients with cervical cancer since the end of treatment according to HPV 16 lineage*. Lineage A = 121 cases, B/C = 12 cases, D = 59 cases. *HPV 16 lineages B and C were grouped due to their low frequency.

Disease-free survival was studied in the 192 women, who did not have persistent disease after treatment. Up to 25% of these patients exhibited a disease-free timeframe lower than 29.6 months. The Kaplan-Meyer disease-free survival curves according to HPV 16 lineage are presented in Fig 3. The median survival time for the second quartile was 29.6 months among patients carrying lineage A, 54.4 months among those carrying the B/C lineages, and 25.4 months among those carrying lineage D. The estimated median was not calculated for group B/C because of the small number of patients carrying these lineages. No significant difference between disease-free survival was found (p = 0.35; log-rank test).

Staging was the only variable significantly associated with the risk of CC recurrence, expressed by risk ratios. In this case, women with advanced staging exhibited an HR of 1.77 (95% CI 1.07–2.92) compared with those with early staging (Table 4).

**Table 4:** Risk of cervical cancer recurrence (n = 192^*^).

Characteristic	n	Crude HR^†^	P value
		(CI 95%)	
**HPV 16 lineage**			
A	121	0.73 (0.44–1.22)	0.23
B/C	12	0.55 (0.19–1.58)	0.27
D	59	1	–
**Age (years)**		1.00 (0.99–1.02)	0.50
**Histological type**			
Squamous cell carcinoma	155	0.64 (0.36–1.14)	0.126
Adenocarcinoma	30	1	–
Others	7	0.31 (0.04–2.36)	0.26
**Staging**			
I or II	130	1	
III or IV	62	1.77 (1.07–2.92)	0.025
**Treatment completion**			
Yes	174	1	–
No	18	1.19 (0.57–2.49)	0.46

* Patients who did not exhibit persistent post-treatment cervical cancer

† univariate cox regression model hazard ratio (HR)

## Discussion

This study aimed to assess the influence of HPV 16 strains on CC prognosis. In this context, age, histopathological type, disease staging, and treatment completion were associated with a higher risk of death.

No associations between HPV 16 lineages and CC prognosis were noted, although different prevalences of each were detected according to stage, with no confounding effect demonstrated between these variables. The low frequencies of lineages B and C may have contributed to the lack of significant associations noted here. The most frequently detected lineage was the A (Asian-European) lineage. Tornesello *et al.* [21] found that lineage D exhibited a more aggressive behavior in CC cases among Italian women. On the other hand, we found an association between lineage D and women that did not complete the treatment.

Although no significant CC prognosis differences were detected in relation to HPV 16 lineages, other authors have noted this association. Zuna *et al.* [23], for example, reported lower survival rates in women carrying the European lineage. In another study, Rader *et al.* [22] reported lower disease-free survival rates in patients carrying the HPV16 B lineage. In our study, the better survival rates observed for patients carrying non-European lineages (B, C and D) were partially mediated by initial staging at diagnosis, suggesting that this lineage is associated with less aggressive tumor behavior compared with the European lineage.

In this study, the median age of patients with CC at diagnosis was 48 years, and a 1% increase in the risk of death was noted with each increasing year. This finding is consistent with those studies that demonstrated age as an independent CC prognostic factor [37–42].

Squamous cell carcinoma was the most common histopathological type detected in the present study, representing 82.3% of all cases, in line with other literature reports [1]. The “others” histopathological category (poorly differentiated carcinoma, adenosquamous, neuroendocrine and sarcoma) presented a higher risk of death compared with adenocarcinomas. These tumors exhibit a more aggressive clinical evolution and are generally diagnosed at more advanced stages [43,44].

In contrast to other authors, who reported worse prognosis for adenocarcinoma cases [45–48], this study did not show a significant difference in risk of death. Interestingly, this may be because most of the adenocarcinoma cases reported herein were detected in stage I.

Staging was the only significant variable associated with CC recurrence risk, which is consistent with the findings of other authors [1,49]. Furthermore, patients exhibited a 4-fold increased risk of death when they did not complete the treatment. Most women unable to complete treatment presented advanced CC stages and exhibited associated comorbidities, such as anemia and kidney failure, making it impossible to complete initial therapeutic plans. Other authors have associated treatment completion and other variables with age, reporting that CC patients aged 70 years or older exhibit a higher rate of less aggressive treatment or were unable to receive treatment at all, although they do not mention why no treatment or incomplete treatment took place [37].

An unequal incomplete treatment distribution according to HPV 16 lineage was noted in the present study, where just over half of the patients carrying lineages A and D did not complete the treatment, whereas 80% of those carrying the B/C lineages did so. The lineage staging distribution may have influenced this result.

Although no significant association between HPV 16 strains and CC prognosis was observed, this relationship has been infrequently evaluated in prior studies. The lack of detection of such an association in the current study may be attributed to the limited sample size for specific HPV 16 lineages.

## Conclusion

No statistically significant associations between HPV 16 lineages and CC prognosis were observed. Age, poorly differentiated carcinoma, adenosquamous, neuroendocrine and sarcoma histopathological types, advanced staging, and incomplete treatment were associated with worse CC prognoses. Considering the sample size limitations of some HPV16 strains in this study, their influence on CC prognosis cannot be disregarded, and further studies in this regard may contribute to elucidate this issue.

## Supporting information

S1 Data Database of patients diagnosed with cervical cancer associated with HPV-16 infection. (XLS)
